# Process development for the continuous production of heterologous proteins by the industrial yeast, *Komagataella phaffii*

**DOI:** 10.1002/bit.26846

**Published:** 2018-10-24

**Authors:** Ayca Cankorur‐Cetinkaya, Nathalie Narraidoo, Ceyda Kasavi, Nigel K.H. Slater, David B. Archer, Stephen G. Oliver

**Affiliations:** ^1^ Department of Biochemistry Cambridge Systems Biology Centre, University of Cambridge Cambridge United Kingdom; ^2^ School of Life Sciences, University of Nottingham, University Park Nottingham United Kingdom; ^3^ Department of Chemical Engineering & Biotechnology University of Cambridge, Cambridge University West Site Cambridge United Kingdom

**Keywords:** antibody, continuous bioprocessing, human lysozyme (HuLy), *Komagataella phaffii* ( *K. phaffi*), *Pichia pastoris* ( *P. pastoris*), process optimization

## Abstract

The current trend in industrial biotechnology is to move from batch or fed‐batch fermentations to continuous operations. The success of this transition will require the development of genetically stable production strains, the use of strong constitutive promoters, and the development of new medium formulations that allow an appropriate balance between cell growth and product formation. We identified genes that showed high expression in *Komagataella phaffii* during different steady‐state conditions and explored the utility of promoters of these genes (Chr1–4_0586 and FragB_0052) in optimizing the expression of two different r‐proteins, human lysozyme (HuLy), and the anti‐idiotypic antibody fragment, Fab‐3H6, in comparison with the widely used glyceraldehyde‐3‐phosphate dehydrogenase promoter. Our results showed that the promoter strength was highly dependent on the cultivation conditions and thus constructs should be tested under a range of conditions to determine both the best performing clone and the ideal promoter for the expression of the protein of interest. An important benefit of continuous production is that it facilitates the use of the genome‐scale metabolic models in the design of strains and cultivation media. In silico flux distributions showed that production of either protein increased the flux through aromatic amino acid biosynthesis. Tyrosine supplementation increased the productivity for both proteins, whereas tryptophan addition did not cause any significant change and, phenylalanine addition increased the expression of HuLy but decreased that of Fab‐3H6. These results showed that a genome‐scale metabolic model can be used to assess the metabolic burden imposed by the synthesis of a specific r‐protein and then this information can be used to tailor a cultivation medium to increase production.

## 1 INTRODUCTION

Recombinant protein production is now a multibillion‐dollar market, and about 25% of commercial pharmaceuticals are biopharmaceuticals (Weinacker et al., 2013). The ever‐increasing demand for therapeutic antibodies may force many companies to change from batch processing to continuous production, because traditional fed‐batch manufacturing cannot generate the huge quantities of r‐proteins required without huge capital investments in a new plant (Warikoo et al., 2012). In addition to increasing demand, the desire for reduced processing costs and requirements for consistent quality and higher productivity are among the key drivers for this transition from batch to continuous in the biotechnology industry (Rathore, Agarwal, Sharma, Pathak, & Muthukumar, 2015).

The switch from batch to continuous processing requires the implementation of quality‐by‐design principles and new product development processes (Hernandez, 2015). On the other hand, short process development times, low tolerance to risk, and cost control under stringent quality/regulatory requirements make the implementation of new technologies a challenge for the biotechnology companies (Rathore et al., 2015). Thus, the development of new approaches and techniques that will reduce the risk and time required for upstream process development and optimization is essential. For a continuous process, an important consideration during the development of novel cell lines and growth media should be to generate stable and robust processes that are able to maintain high productivity over a considerable period of continuous operation.

The choice of a vehicle organism for r‐protein production is often determined by the nature of the recombinant product, with mammalian systems being preferred for the production of large, glycosylated proteins, and microbial cells for smaller unmodified proteins. Recent advances in the humanization of the microbial pathways for protein glycosylation and the burgeoning demand for continuous processes make microbial eukaryotes and particularly yeasts increasingly attractive vehicles for the production of complex human proteins (Maccani et al., 2014). Among the yeasts, *Komagataella phaffii* (formerly known as *Pichia pastoris*) has been the species most often used for the production of biopharmaceuticals and industrial enzymes. This is due to the ability of *K. phaffii* to grow to very high cell densities, the availability of strong and tightly regulated promoters, its ability to secrete high titers of properly folded after being translationally processed, and active recombinant proteins, as well as the recent availability of engineered strains that are able to mimic the human protein glycosylation pathways (Ahmad, Hirz, Pichler, & Schwab, 2014). AOX is a strong methanol‐inducible promoter that is widely used for transgene expression in *K. phaffii*. However, although this promoter is extensively used for fed‐batch processes, it is not suitable for the continuous production of recombinant proteins. A number of constitutive promoters for this yeast has been reported, although most have been tested only in batch and fed‐batch systems (Prielhofer et al., 2013; Stadlmayr et al., 2010; Vogl & Glieder, 2013); among these, the glyceraldehyde‐3‐phosphate dehydrogenase promoter (GAP) was reported as giving strong expression and has been the most often exploited (Vogl & Glieder, 2013). For all this, the identification of strong, constitutively expressed, and promoters of proven utility in continuous fermentations remains an important goal.

The development and optimization of the growth medium and physical operating parameters are key factors in the achievement of high product yields with the required quality specifications (Kunert & Reinhart, 2016). Leading suppliers and recombinant protein manufacturers have performed a lot of research work in media optimization for mammalian cell lines on an individual basis for each process and cell line, in some cases resulting in >100‐fold improvements in product yields (Bonander et al., 2009). On the other hand, most of the studies reported in the literature on *P. pastoris* (*K. phaffii*) that compare the strength of different promoters in this host fail to optimize the strain or the culture conditions to optimize the performance of each promoter. The medium compositions most commonly used for high cell‐density fermentations by *P. pastoris* (*K.phaffii*) are the following: a basal salt medium proposed by Invitrogen (2000), FMM22 formulated by Higgins, Cregg, Stratton, Chiruvolu, & Meagher (1998), and another alternative developed by D’Anjou & Daugulis (2000). All these media were formulated to obtain high cell densities in fed‐batch cultures (Cos et al., 2006). A recent review on r‐protein production by *K. phaffii* using the GAP promoter summarized studies investigating the effect of different carbon sources, or amino‐acid supplementations, different bioreactor operation parameters (i.e., pH, temperature, oxygenation level) on the quality and quantity of the r‐protein production (Çalık et al., 2015). The fact that most of these studies investigated one process parameter at a time and the concentrations of other nutrients were generally kept constant across different studies implies that there remains a room for improving productivity levels by further media development using multiparametric optimization (Cankorur‐Cetinkaya, Dias et al., 2017).

In this study, we have investigated the potential of new promoters for constitutive expression of the r‐proteins using *K. phaffii* as the host organism. We explored the effect of medium composition on strain performance and showed its importance in the identification of the most productive strain. We have also investigated the effect that the identity of r‐protein to be produced and the promoter from which its cognate transgene is expressed has on the development of an optimal growth medium. Moreover, in a test case, we have shown how model‐based approaches can be used to tailor the growth medium to optimize the production of a specific r‐protein.

## 2 MATERIALS AND METHODS

### 2.1 Strain construction and verification

The native *K. phaffii* promoters of interest were amplified from the genomic DNA of *K. phaffii* X‐33. The light and heavy chain fragments of Fab‐3H6 were amplified by polymerase chain reaction (PCR) from the vector pGAPZ⍺A+3H6, kindly provided by Diethard Mattanovich. The strains expressing either human lysozyme (HuLy) or the anti‐idiotypic antibody fragment, Fab‐3H6 were constructed as described in the Supporting Information File 1.

### 2.2 Cultivations

Precultures of each clone were prepared in yeast extract‐peptone‐glycerol with a single colony selected from yeast extract, peptone, agar, glycerol (YPAG) plates. The precultures were grown, with shaking at 200 rpm, for ca., 24 hr at 30°C to an approximate optical density (OD_600_) of 15–25, and used to inoculate the main cultures to an OD_600_ of 0.05. For strain characterization, cells were cultivated in complex (10 g/L yeast extract, 10 g/L peptone, 13.4 g/L YNB with ammonium sulfate, 100 mM potassium phosphate buffer at pH 6 and 0.4 mg/L biotin, 40 g/L glycose or glycerol), rich (10 g/L yeast extract, 20 g/L peptone, 40 g/L glycose or glycerol) or minimal media described as the batch medium (either using glucose or glycerol as the carbon source) by Prielhofer et al. (2013). The effect of addition of sorbitol as an additional carbon source as well as characterization of the Fab‐3H6 producing clones and stability tests were performed using the minimal medium. Chemostat experiments to test the stability of the strains were performed in 2 L fermenters with a working volume of 1 L and 0.1 hr^−1^ dilution rate (unless otherwise stated) using the chemostat medium (best‐performing condition; BPC) described by Baumann et al. (2008). Cultures were first grown overnight in batch mode (30°C; 750 rpm stirrer speed; aeration with 1 L·min^−1^ air) before switching to chemostat cultivation. Cells were tested under 12 different medium compositions (Supporting Information File 2) using Micro‐Flasks (Duetz system, Adolf Kuhner AG, Basel, Switzerland) with 96‐deep‐well plates and a sandwich cover. Citrate‐phosphate buffer was used to keep the pH at 6. Optimal conditions to prevent the formation of precipitates in the media were found to be 500 μl working volume at 350 rpm in an orbital shaker providing horizontal plane rotary motion in a 1” (2.54 cm) circular orbit. Cells were grown in 250 ml baffled shake flasks with a working volume of 50 ml at 200 rpm to compare their growth characteristics under the BPC among the 12 tested. Model verification experiments were conducted in 96‐deep‐well plates as described above. The amino‐acid supplementations were done such that tryptophan and phenylalanine concentrations were 3 mM and the tyrosine concentration was 2.5 mM (due to its lower solubility). The effect of tyrosine supplementation was also confirmed in chemostat experiments using the BPC with or without tyrosine. For chemostat experiments, the pH of the feed was set to 5 to prevent any precipitation. BPC medium composition: 11.52 g/L (NH_4_)_2_PO_4_, 0.61 g/L KCl, 2.37 g/L MgSO_4_·(H_2_O)_7_, 0.1 g/L FeSO_4_·(H_2_O)_7_, 0.05 g/L CaCl_2_·(H_2_O)_2_, 25.32 g/L glucose, 10.65 g/L sorbitol, 4 g/L citric acid, 4.6 ml/L PTM1 trace salt solution and 0.45 g/L tyrosine (under+tyr conditions). PTM1 trace salts stock solution contained per liter: 6.0 g CuSO_4_·(H_2_O)_5_, 0.08 g NaI, 3.36 g MnSO_4_·H_2_O, 0.2 g Na_2_MoO_4_·(H_2_O)_2_, 0.02 g H_3_BO_3_, 0.82 g CoCl_2_, 20.0 g ZnCl_2_, 0.2 g biotin, and 5.0 ml H_2_SO_4_ (95%–98%). Chemostat experiments were performed in 2 L fermenters with a working volume of 1 L and 0.1 hr^−1^ dilution rate. Cultures were first grown overnight in batch mode (30°C; 700 rpm stirrer speed; aeration with 1 L·min^−1^ air) before switching to chemostat cultivation. The pH was kept constant at 5 by the regulated dosing of 0.5 M sodium hydroxide.

### 2.3 R‐protein quantifications and metabolite analysis

HuLy activity in culture supernatants was quantified using the EnzChek Lysozyme Assay Kit (Molecular Probes, Invitrogen Detection Technologies, Thermo Fisher Scientific, Waltham, MA) according to the manufacturer’s instructions. Fab‐3H6 was quantified by sandwich ELISA using Goat AntiHuman IgG Fd gamma (ab79108) as the capture antibody and Goat AntiHuman IgG F(ab; ab49761) as the secondary antibody. Using the clones expressing only light or heavy chain fragments of Fab‐3H6 as the negative control, it was confirmed that, using this antibody pair, only the intact form of the Fab‐3H6 is quantified but not the free light or heavy chains.

Extracellular metabolite concentrations were determined enzymatically (R‐Biopharm; Darmstadt, Germany) Yellow Line Enzymatic BioAnalysis and Food Analysis Kits (Cat no: 10716251035 sucrose/d‐glucose, 10148270035 glycerol, 10670057035 sorbitol, 11112732035 ammonia) as described by the manufacturer. The dry weight was determined gravimetrically. Significance analyses between the utilization of the substrates and r‐protein production levels were performed using Student’s *t* test.

### 2.4 Flux balance analyses using the genome‐scale metabolic model

The Kp. 1.0 stoichiometric model of the *K. phaffii* metabolic network (Cankorur‐Cetinkaya, Dikicioglu, & Oliver, 2017) was used in flux balance analysis (FBA). Reactions for the synthesis of lysozyme were incorporated into the model (Kp. 1.1; Supporting Information File 3). Analyses were conducted using the COBRA tool‐box (v.2.0.5) under MATLAB R2013b (8.2.0.701 Mathworks; Natick, MA; Schellenberger et al., 2011) with SBML Toolbox v4.1.0 and libSBML library v5.5.0 using the Gurobi5 solver. The simulations were conducted by constraining the glucose and sorbitol uptake rates to unity with an objective function of maximization of growth. The simulations of r‐protein production were performed separately for each protein by constraining the production reaction of the corresponding r‐protein to 0.05, while constraining the production reaction of the other r‐protein to zero. The significantly changing fluxes between r‐protein‐producing transformants and wild‐type were assessed by using both the Mann–Whitney U test (*p* < 0.01) and fold change (FC > 1.5) analyses, as described previously (Cankorur‐Cetinkaya, Dikicioglu et al., 2017) and in Supporting Information File 1. The lists of reactions that were identified to be significantly and differentially changed and associated with higher flux values with increased r‐protein production, and the list of genes associated with those reactions are provided in Supporting Information File 4. For the simulations, the maximization of the r‐protein production was used as the objective function, the growth rate was set to 0.25 hr^−1^ and glucose and sorbitol uptake rates were set to unity as before. Gene ontology (GO)‐term enrichment analysis was conducted as described previously (Cankorur‐Cetinkaya, Dikicioglu et al., 2017).

## 3 RESULTS

### 3.1 Identification of new promoters

Previously, we had studied the genome‐wide gene expression changes both before and after the induction of a transgene under the control of the AOX promoter in chemostat culture. The r‐proteins expressed were native lysozyme and two mutants (Kumita et al., 2006) designed to induce misfolding of the r‐protein: one, an amyloidogenic variant (I56T), prone to intracellular aggregation and the other a variant (T70N) that results in a protein that is misfolded but remains secretable (Hesketh, Castrillo, Sawyer, Archer, & Oliver, 2013). The expression data from these experiments were used to identify the genes that are highly expressed in all steady‐state conditions. Two genes, Chr1–4_0586 and FragB_0052, were identified as being in the top five most expressed genes in both the preinduction and postinduction steady states across all three strains (Supporting Information File 5). The function of the protein encoded by Chr1–4_0586 is unknown, but is orthologous to the *SPI1* gene in *Saccharomyces cerevisiae* (Valli et al., 2016) and FragB_0052 encodes translational elongation factor EF‐1 alpha (TEF1‐⍺). The promoter of TEF1‐⍺ was previously shown to have a strong promoter activity capable of producing r‐proteins at levels similar to or higher than those produced by the GAP promoter in fed‐batch cultures (Ahn et al., 2007).

### 3.2 Strain characterization

The relative efficiencies of three constitutive promoters (GAP, TEF1‐⍺, and oSPI1 – ortholog of *SPI1*) were assessed in the optimization of the expression of two different r‐proteins: HuLy and Fab‐3H6 (an anti‐idiotype to an HIV‐neutralizing antibody). To ensure that this comparison was fair, it was essential that each of the transgenes was inserted at the same site in the *K. phaffii* genome and was present at the same copy number. Therefore, only constructs in which a single copy of the transgene had integrated into its cognate promoter locus in the *K. phaffii* genome were included in the study. After screening more than hundred constructs for each case, five independent single‐copy clones expressing HuLy under the control of the oSPI1 promoter and six using the GAP promoter, as well as two different clones expressing Fab‐3H6 under the TEF1‐⍺ or the GAP promoter were identified and subjected to further evaluation.

**Figure 1:**
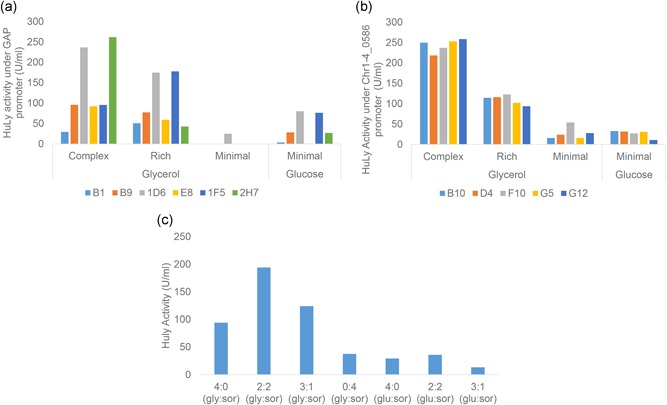
Characterization of the HuLy producing strains. This figure shows the secreted HuLy levels by (a) six different clones expressing it under GAP promoter and (b) five different clones expressing it under oSPI1 promoter, under four different medium compositions. (c) The effect of sorbitol addition on the secreted HuLy levels was investigated when it was expressed under the oSPI1 promoter using Clone F10. GAP: glyceraldehyde‐3‐phosphate dehydrogenase promoter; HuLy: human lysozyme

**Figure 2:**
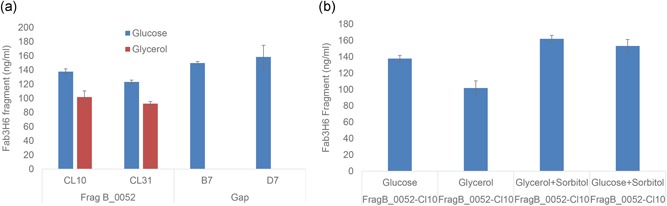
Characterization of Fab‐3H6 producing strains. (a) The figure shows the secreted Fab‐3H6 concentration when it was expressed under the TEF1‐⍺ using either glucose or glycerol as the carbon source or under GAP promoter when glucose was used as the carbon source. (b) The effect of sorbitol addition on the secreted protein levels was investigated when it was expressed under the TEF1‐⍺ promoter using the Clone 10. GAP: glyceraldehyde‐3‐phosphate dehydrogenase promoter; TEF1‐⍺: translational elongation factor EF‐1 alpha

To identify the clones yielding the highest protein titers, the HuLy‐producing clones were grown in complex, rich, and minimal media using either glucose or glycerol as the carbon source. The clone that performed best on average across all conditions was determined for each promoter and these (clones 1D6 and F10 expressing HuLy under the control of the GAP and oSPI1 promoters, respectively) were used for further studies (Figure 1a,b). In the case of Fab‐3H6 producing clones, because glucose was previously reported as the preferred carbon source for the expression of this antibody under the control of the GAP promoter (Buchetics et al., 2011), these clones were tested only using glucose as the carbon source; whereas the clones expressing Fab‐3H6 under the control of the TEF1‐⍺ promoter were tested using both glycerol and glucose as carbon sources (Figure 2a). We found that clonal variation between the Fab‐3H6‐producing integrants was less than that found between the different HuLy‐producing strains. Nevertheless clones that produced slightly higher titers of Fab‐3H6 than average (Cl10 and B7) were selected for further study.

This initial set of experiments also allowed us to identify the preferred carbon source for each clone. Glucose was identified as the preferred carbon source for the expression of HuLy under the control of the GAP promoter and of Fab‐3H6 using the TEF1‐⍺ promoter, whereas glycerol was the preferred carbon source for the expression of HuLy under the control of the oSPI1 promoter. We further investigated the effect on the r‐protein titers of using sorbitol either as a sole carbon source or as a dual carbon source together with either glycerol or glucose. It was observed that using the sorbitol in 1:1 ratio with glycerol resulted in a twofold increase in the product titer in the case of HuLy expression under the control of the oSPI1 promoter (Figure 1c). In the case of Fab‐3H6 expression under the control of the TEF1‐⍺ promoter, addition of sorbitol as a dual carbon source together with either glycerol or glucose in 1:1 ratio improved r‐protein production, resulting in similar product titers for both carbon sources (Figure 2b).

**Figure 3:**
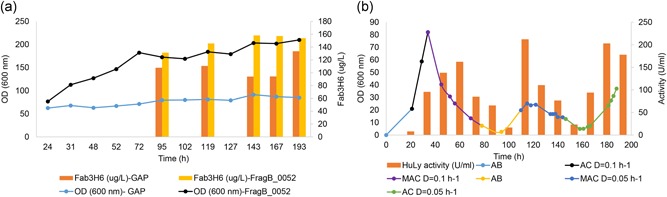
R‐protein production in a continuous fermentation. (a) Fab‐3H6 producing clones under GAP and TEF1‐⍺ promoters were cultivated in a chemostat mode around 200 hr and steady state Fab‐3H6 levels were measured. (b) HuLy producing strain under GAP promoter was cultivated in a continuous mode under different aeration rates and dilution rates. AB: aerated‐batch; AC: aerated continuous; D: dilution rate; GAP: glyceraldehyde‐3‐phosphate dehydrogenase promoter; HuLy: human lysozyme; MAC: microaerated continuous; OD: optical density; TEF1‐⍺: translational elongation factor EF‐1 alpha

To test the stability and monitor the productivities of the clones when cultivated in a continuous mode, a set of chemostat experiments were performed. It was verified that the clones under investigation express the r‐protein and remain stable for at least 30 generation (200 hr of chemostat experiment with a dilution rate of 0.1 hr^−1^). It was observed that the clones expressing Fab‐3H6 readily achieved a steady state when the previously reported condition for fully aerobic chemostat experiments were used (Baumann et al., 2008). It was also observed that the clone expressing Fab‐3H6 under the control of the TEF1‐⍺ promoter yielded higher protein titers than one with the GAP promoter (Figure 3a).

The high levels of proteins secreted by the HuLy‐producing strains, led to excessive foam formation, which could not be countered by the use of antifoam and prevented the observation of steady‐state growth. Attempts to prevent foam formation by reducing aeration compromised the growth rate and led to wash out. Nevertheless, samples collected from cultures grown under all these conditions showed that there was no loss in cell viability and that r‐protein production was maintained (Figure 3b). We chose the clone that expressed HuLy under the control of the oSPI1 promoter for additional chemostat experiments with either an increase in the dilution rate or a reduction in the concentration of the carbon source in the growth medium. It was observed that, when the dilution rate was increased from 0.1 to 0.2 hr^−1^, the excessive foam formation problem was solved, but the productivity decreased. On the other hand, when the carbon source’s concentration was decreased from 40 to 20 g/L glycerol, the foaming problem was solved without any reduction in the volumetric productivity of HuLy (Table 1).

**Table 1:** Comparison of the productivity levels of HuLy‐producing strain using the oSPI1 promoter

Dilution rate	Carbon source (g/l)	HuLy activity (U/ml)	HuLy productivity (U/ml/h)	Foaming
0.1	40	63.33	6.33	Excessive
0.2	40	35.00	7.00	Normal
0.1	20	74.17	7.42	Normal

*Note*. HuLy: human lysozyme

### 3.3 Effect of cultivation conditions on strain performance

**Figure 4:**
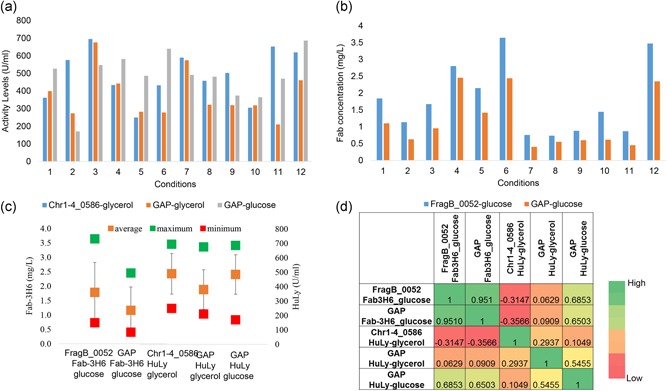
R‐protein production across different conditions. The strains producing HuLy and Fab‐3H6 were cultivated under 12 different conditions and the secreted r‐protein levels were compared. (a) Secreted HuLy activity levels at stationary phase across 12 conditions by cells expressing HuLy under oSPI1 promoter when glycerol used as the carbon source (blue), under GAP promoter when glycerol or glucose was used as the carbon source (orange and gray, respectively). (b) Secreted Fab‐3H6 concentrations at stationary phase across 12 conditions by the cells expressing antibody under the TEF1‐⍺ (blue) and GAP (orange) promoters when glucose was used as the carbon source. (c) The green and red squares represent the maximum and minimum level of r‐protein concentrations observed across 12 tested conditions, respectively. The orange square represents the average of the protein level attained in those 12 conditions. Error bars represent the variation across the conditions. (d) Spearman correlation coefficient values between the rankings of the conditions from high to low level of r‐protein between different strains. GAP: glyceraldehyde‐3‐phosphate dehydrogenase promoter; HuLy: human lyzozyme; TEF1‐⍺: translational elongation factor EF‐1 alpha

Preliminary experiments indicated that it would be necessary to determine the optimum cultivation conditions for each r‐protein/promoter combination used. Accordingly, we tested all the strains under consideration (the HuLy‐producing strains using either the GAP or oSPI1 promoters and the Fab‐3H6‐producing strain using either the GAP or TEF1‐⍺ promoters) under 12 different conditions. These conditions were randomly generated using CamOptimus (Cankorur‐Cetinkaya, Dias et al., 2017) by changing the concentrations of seven nutrients: ammonium, potassium, magnesium, iron, calcium, sorbitol, and glucose (for TEF1‐⍺ and GAP promoters) or glycerol (for the oSPI1 promoter). The performance of the strains was evaluated by comparing the r‐protein levels obtained under the 12 different conditions (Figure 4). To control any effect of the carbon source, the HuLy‐producing strain using the GAP promoter was evaluating on both glucose and glycerol as the carbon source.

The comparison of the strains under these conditions revealed that, for HuLy production by glycerol‐grown cells, the oSPI1 promoter outperformed the GAP promoter in 8/12 of the conditions tested (Figure 4a). In 9/12 of the conditions tested, the production of HuLy using the GAP promoter was higher in glucose‐grown cells than in glycerol‐grown ones (Figure 4a). These results contrasted with those for Fab‐3H6 production by glucose‐grown cells, where the TEF1‐⍺ outperformed GAP in all conditions tested (Figure 4b).

Optimization of promoter/growth conditions is clearly important because there is a sixfold range in r‐protein yields across the different conditions (Figure 4c). However, some general trends are discernible; for instance, the levels of secreted Fab‐3H6 protein were higher in all conditions when the TEF1‐α, rather than the GAP, promoter was used. When we ranked the r‐protein yield for each clone across the different conditions and computed the Spearman’s rank correlations between these rankings (Figure 4d), it was observed that the change in performance of Fab‐3H6‐producing strains was significantly similar to each other. The ranking of the conditions for the HuLy‐producing strain using the GAP promoter with glucose as the carbon source was also significantly similar those of the Fab‐producing strains (*p* < 0.05). On the other hand, the performance of the HuLy‐producing strains under oSPI1and GAP did not appear to be correlated across the tested conditions. There were also no correlations between the rankings of the conditions between antibody‐producing strains and the HuLy‐producing strain using the oSPI1 promoter. These results indicate that, despite some recognizable trends within data sets, no general inference can be made concerning how cells expressing the same r‐protein using different promoters or different r‐proteins using the same promoter will perform across different conditions. It is evident that the development of a widely usable promoter/condition combination will require a large number of experiments (including directed mutation of promoter sequences; Hartner et al., 2008) and the application of a machine‐learning approach (Cankorur‐Cetinkaya, Dias et al., 2017) to this multiparametric optimization problem, if the number of trials involved is not to become unfeasibly large.

### 3.4 Metabolic modeling to tailor the cultivation conditions

When the conditions were ranked based on their r‐protein yields, condition 12 was the only one that proved to lie within the 3 best‐performing conditions for all strains. The performance of these four strains expressing two different r‐proteins under two different promoters was further studied under this BPC together with the wild‐type strain as the control case. We found that expression of either of the two different r‐proteins did not result in any significant change in substrate consumption levels with respect to wild‐type cells at the mid‐exponential phase (*p* > 0.01) and there was no residual glucose or glycerol remaining at the stationary phase, indicating that the cultures were glucose (or glycerol) limited (Supporting Information File 6). Given the similarity of these gross indicators of performance, we decided to investigate the effect of expressing two different r‐proteins on the metabolic flux distribution by performing simulations in silico.

The reactions for the HuLy synthesis were incorporated into the genome‐scale metabolic model of *K. phaffii* (Cankorur‐Cetinkaya, Dikicioglu et al., 2017; reactions for Fab‐3H6 were already in the model). This version (Kp.1.1) was used to identify the fluxes that show significant change, with respect to wild‐type cells, when the transformants express either HuLy or Fab‐3H6. The simulations were conducted with an objective function of maximization of the growth rate by using the same constraints for carbon source uptake rates for all strains. This is appropriate because no significant difference was observed in the glucose and sorbitol consumption between wild‐type and recombinant strains at mid‐exponential phase, which is analogous to steady growth. The uptake rates of the other substrates were not constrained as the tested condition was carbon limited. No fewer than 27 reactions were identified to be significantly (*p* < 0.01) and differentially (FC > 1.5) changed between the Fab‐3H6‐producing and the wild‐type strains. To identify the reactions that were associated with higher fluxes as the r‐protein production reaction was constrained with higher values, the flux scanning based enforced objective function (Choi, Lee, Kim, & Woo, 2010) was used. The reactions that are both associated with higher flux values as the r‐protein production were increased, and showed significant change in comparison with wild‐type simulations that were identified. In the case of the Fab‐3H6‐producing strain, there were 21 reactions associated with 15 genes. These genes were significantly enriched for annotations associated with the chorismate and tyrosine biosynthetic pathways (*p* < 0.05). In the case of Huly production, there were 26 reactions satisfying these conditions and these reactions were associated with 21 genes whose annotations were significantly enriched with tryptophan and chorismate metabolic processes terms. The simulations were also conducted with an objective of maximization of r‐protein production by using the same constraints for substrate uptake and biomass production rates for the HuLy and the Fab‐3H6‐producing strains. A total of 23 reactions that were associated with 13 genes, were identified to be significantly (*p* < 0.01) and differentially (FC > 1.5) changed between Fab‐3H6‐producing and HuLy‐producing strains. These genes were determined to be significantly enriched for annotations using the GO process terms “tryptophan metabolic process” and “sulfate assimilation process” (*p* < 0.01).

**Figure 5:**
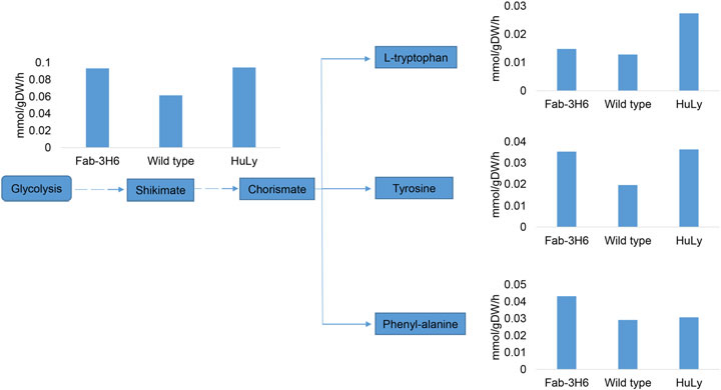
In silico flux distribution through the aromatic amino acid pathway. The bar graphs indicate flux values going through each of these linear pathways when simulations were done for the expression of Fab‐3H6, HuLy or wild‐type case. HuLy: human lyzozyme

These analyses indicated that the in silico fluxes through aromatic amino‐acid biosynthesis were increased by r‐protein production. Examination of the in silico flux values for this pathway revealed that the fluxes going through shikimate and tyrosine biosynthesis pathways were higher in the both types of recombinant cells in comparison to the simulations with wild‐type. Additionally, fluxes going through tryptophan biosynthesis in HuLy‐producing cells were higher than both wild‐type and Fab‐3H6‐producing cells. Similarly, fluxes going through phenylalanine biosynthesis were higher in Fab‐3H6‐producing cells than the others (Figure 5).

**Figure 6:**
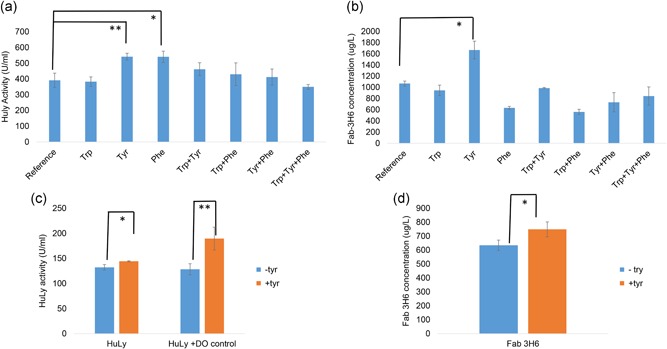
Effect of aromatic amino acid supplementation on r‐protein production. This figure represents the secreted (a) HuLy activity and (b) Fab‐3H6 concentration under reference and amino acid supplemented conditions. The effect of tryptophan (Trp), tyrosine (Tyr), phenylalanine (Phe), tryptophan and tyrosine (Trp+Tyr), tryptophan and phenylalanine (Trp+Phe), tyrosine and phenylalanine (Tyr+Phe), and tryptophan, tyrosine and phenylalanine (Trp+Tyr+Phe) supplementations were investigated using the strains expressing these r‐proteins under the GAP promoter. The effect of tyrosine supplementation (+tyr) was tested in chemostat cultures and compared with cases where no supplementation was applied (−tyr) with strains producing (c) HuLy or (d) Fab‐3H6 using the GAP promoter. **represents the significance level below 0.01 and *represents the significance level below 0.05, +DO indicates the conditions where, DO levels were kept above 50%. DO: dissolved oxygen; GAP: glyceraldehyde‐3‐phosphate dehydrogenase promoter; HuLy: human lyzozyme

To test the effect of supplementation of aromatic amino acids on protein titers, HuLy‐ and Fab‐3H6‐producing strains, both using the GAP promoter were tested under eight different conditions: (a) reference, (b) tryptophan supplementation, (c) tyrosine supplementation, (d) phenylalanine supplementation, (e) tryptophan and tyrosine supplementation, (f) tryptophan and phenylalanine supplementation, (g) tyrosine and phenylalanine supplementation, and (h) supplementation with all three aromatic amino acids. Both tyrosine and phenylalanine were identified to result in a significant (*p* < 0.05) increase in the secreted HuLy levels when they were supplemented individually (Figure 6a). The secreted HuLy levels were around 40% higher in comparison to the reference case, where no amino‐acid supplements were provided. On the other hand, addition of both amino acids at the same time, or these two together with tryptophan, did not cause any significant change in the secreted protein levels as compared with the reference condition. Tyrosine supplementation also resulted in a significant (*p* < 0.05) increase in the secreted antibody concentration (Figure 6b). Fab‐3H6 concentrations in the tyrosine‐supplemented cultures were around 55% higher than that of the reference cultures. However, supplementation with phenylalanine alone or together with tryptophan resulted in significant (*p* < 0.01) decreases in the antibody titers (40% and 47%, respectively).

The effect of the tyrosine supplementation, which was identified to improve both HuLy and Fab‐3H6 titers in batch cultures, was further tested in continuous fermentations. For this purpose, chemostat experiments were conducted with strains producing either HuLy‐ or Fab‐3H6, using the GAP promoter, with and without tyrosine supplementation. The protein titers were determined after the cultures had spent three, five, and seven residence times at steady state (constant cell density and dissolved oxygen [DO] levels). The protein levels were significantly higher under tyrosine‐supplemented conditions (*p* < 0.05). In the case of Fab‐3H6 production, r‐protein levels were around 20% higher in the condition with tyrosine supplementation, as compared with the case without tyrosine supplementation (Figure 6d). In the case of HuLy production, the tyrosine‐supplemented condition yielded 9% higher r‐protein levels compared with the case without tyrosine (Figure 6c). In both cases, DO levels were lower when tyrosine was supplemented, indicating a higher oxygen demand when this aromatic amino acid was supplied (Supporting Information File 7). In the case of HuLy expressing conditions, DO levels decreased to around zero under tyrosine‐supplemented conditions, whereas DO was at ca., 50% without tyrosine supplementation. As this indicated that oxygen might be limiting under tyrosine‐supplemented conditions, the DO level was controlled such that it did not fall below 50% after cells had spent seven residence times under aerated conditions. The experiments were continued for an additional seven residence times and samples were collected after the cells spent three, five, and seven residence time under DO‐controlled conditions. The supply of additional oxygen resulted in a significant increase of cell density (*p* < 0.01) and increased the r‐protein levels by 30% when tyrosine was provided, indicating that the system had been oxygen limited (Figure 6c). Under conditions where the DO level was controlled, the Huly levels were 46% higher when tyrosine was supplemented compared with conditions without tyrosine supplementation.

## 4 DISCUSSION

In this study, we have exploited the transcriptome data that we previously obtained from chemostat cultures of r‐protein producing strains of *K. phaffii* at different steady states to identify the genes that are always constitutively highly expressed. The promoters of two of these genes were assessed for their utility in optimizing the expression of two different r‐proteins. One of these promoters (that from FragB_0052 or the TEF1‐⍺ gene) had previously been shown to have a strong promoter activity (Ahn et al., 2007), whereas the oSPI1 promoter had not been previously identified as a strong promoter. Both of these constitutive promoters were compared with the frequently used GAP promoter (Vogl & Glieder, 2013).

Any realistic comparison of the potential utility of a novel promoter as compared with that of strong promoters that are already widely used requires many factors to be taken into consideration. It was previously shown that there is large clonal variability between *K. phaffii* transformants to ectopic integration events within this host’s genome (Schwarzhans et al., 2016). Both the genomic context and variation in the copy number of the transgene may contribute to variation in expression levels and so compromise any meaningful comparison of promoter strengths. For these reasons, all the *K. phaffii* transformants that were demonstrated to contain only a single copy of the vector inserted into the chromosomal site of the promoter locus in the correct orientation were used to express the transgene. Despite these precautions, although all selected transformants had a uniform morphology, we observed a high level clonal variation among the clones producing HuLy using the GAP promoter and this may indicate that some undetected chromosomal rearrangements or other genomic changes had occurred (Figure 1a). In addition to these genetic factors, another important parameter that needs to be considered is the growth stage of the inoculum, while comparing different clones. It is important to keep the inoculum volume, age, and lag phase similar between different cultures (Sen & Swaminathan, 2004). Moreover, it is also important to compare the different clones under a range of cultivation conditions. This proved especially important in the case of the HuLy‐producing clones that used the GAP promoter, where we recorded large changes in performance between the complex, rich, and minimal media. The fact that there was no detectable secreted protein for some clones when grown in minimal medium might also indicate some unidentified mutation that occurred during strain construction.

One of the biotechnology industry’s main concerns about switching to continuous fermentations is the possibility that the genetic instability of the recombinant strains may limit the length of production runs (Hesketh et al., 2013); this concern is particularly acute for *K. phaffii* strains, where clonal variation is a particular problem. Therefore, it is essential to test the stability of the clones before any further process optimization. We confirmed the stability of our clones by testing the productivity of the clones in chemostat cultivations of ca., 200 hr, either at steady state or under conditions where the growth rate or aeration rate were periodically altered.

Even having selected the best‐performing single‐copy integrant at the correct locus, comparison of promoter strengths is still problematic. Published growth‐medium formulations relate to r‐protein production only using well‐established and widely‐used promoters. By definition, for a novel promoter, there is no prior information about the cultivation conditions that would maximize the promoter’s strength. To avoid any biased comparison, we tested the clones under 12 different conditions by varying the concentrations of the seven nutrients in the minimal defined medium. Although, in the case Fab‐3H6 production, the TEF1‐⍺ promoter outperformed the GAP promoter under all conditions, the oSPI1 promoter yielded higher HuLy levels only under some conditions. This indicates that the choice of an ideal promoter for the expression of HuLy depends on the cultivation medium, and that both promoters had the potential for high‐level production if the correct conditions were chosen. Furthermore, it was observed that when two different r‐proteins were produced using the same promoter, there was only a weak correlation between performance of the cultivation condition used (Fab‐3H6 and HuLy expression under GAP promoter; Spearman correlation coefficient = 0.65). However, the performance of the Fab‐3H6‐producing strains using two different promoters showed good correlation between r‐protein titers and the various conditions (Spearman correlation coefficient = 0.95). These results show that comparison of promoter strengths under only a single selected condition might lead process developers to discard the optimal promoter for their product, especially if that promoter is a novel one.

Although the performance differences between the clones under different cultivation conditions indicate that these conditions should be optimized in a strain‐specific manner, there was a particular condition under which all clones performed well. Investigation of the growth characteristics of the clones under this condition did not reveal any significant difference in terms of substrate consumption and biomass production. Because there were no gross physiological changes, it was important to assess the impact that r‐protein production might have on the distribution of the fluxes through the metabolic network (Çelik, Çalık, Halloran, & Oliver, 2007), and so we used simulations using the genome‐scale metabolic model to predict any redistribution of the metabolic fluxes. Comparison of the flux distributions highlighted the fact that both HuLy and Fab‐3H6 production resulted in higher net flux values through the aromatic amino‐acid biosynthesis pathway, indicating a metabolic burden associated with this pathway. These model predictions indicated the potential for the further improvement of r‐protein yields by supplementing the growth medium with aromatic amino acids.

Experiments designed to test these model predictions showed that tyrosine supplementation resulted in significant increases in the production of both HuLy and Fab‐3H6. The chemostat experiments performed with tyrosine supplementation also showed the effect of tyrosine on the oxygen demand of the cells, indicating the rewiring of the metabolism under tyrosine‐containing conditions. Intracellular free tyrosine levels were previously reported to increase under oxygen‐limited conditions (Carnicer et al., 2012). These findings may indicate a relationship between tyrosine utilization and oxygen availability, explaining the higher r‐protein levels under oxygen‐rich conditions, as compared with oxygen‐limited conditions when tyrosine was supplemented. It should be noted that tryptophan supplementation had no significant impact on the production of either r‐protein, whereas phenylalanine supplementation had opposite effects on HuLy and Fab‐3H6 expressions (increasing that of the former and reducing that of the latter). Whilst any definitive interpretation of these results awaits detailed metabolomic analyses, it may be proposed that tyrosine inhibition of chorismate mutase (the enzyme at the branch‐point in the aromatic amino‐acid biosynthesis pathway) might channel the chorismate towards tryptophan synthesis and therefore improve HuLy production. Similarly, one can speculate that the increase by tyrosine supplementation and the decrease by phenylalanine supplementation in the Fab‐3H6 levels could be explained by the fact that the presence of phenylalanine inhibits tyrosine synthesis (Mannhaupt, Stucka, Pilz, Schwarzlose, & Feldmann, 1989), and thus the tyrosine levels are the key factors effecting the antibody’s production. Thus, whereas model predictions and the experimental results may point to the genes encoding enzymes in the aromatic amino acid‐biosynthesis as a possible target for genetic engineering, and the end products of this pathway as possible feed supplements, the feedback inhibition of aromatic amino‐acid metabolism involves the fine‐tuning of the internal concentrations of these amino acids and demands that we develop a better understanding of the redistribution of metabolic fluxes in *K. phaffii* cells when nutrient supplements are supplied.

In this study, we have shown how transcriptome data can assist in identifying new promoters that would be suitable for use in the continuous production of r‐proteins. We found that evaluating promoter strength under only one set of cultivation conditions might cause good candidate promoters to be unnecessarily discarded. We also showed that the development of a continuous bioprocess can also benefit from in silico predictions made using metabolic models. In this study, we showed how such predictions can be used to tailor the composition of the growth medium to optimize the production of a specific r‐protein.

## AUTHOR CONTRIBUTIONS

S. G. O, D. B. A, and N. K. H. S conceived the study. A. C. C and S. G .O designed the experiments. A. C. C, N. N, and C. K carried out the experiments. A. C. C carried out the simulations. A. C. C and S. G. O wrote the manuscript. All authors read, revised, and approved the final version of manuscript.

## Supporting information

Supporting information

Supporting information

Supporting information

Supporting information

Supporting information

Supporting information

Supporting information

Supporting information
