# Supplementary material for: Process development for the continuous production of heterologous proteins by the industrial yeast, Komagataella phaffii
Source: Biotechnol Bioeng. 2018 Oct 24;115(12):2962–73. doi: 10.1002/bit.26846 (PMC6283250; doi:10.1002/bit.26846)
Supplement: Supplementary file 1 — Supporting information [file BIT-115-2962-s001.docx]

**Strain Construction**

LC and HC were respectively cloned within the *Eco*R1 and *Not*1 sites of the pGAPZαA vector (Sigma) to generate pGAPZalfaA+HC and pGAPZalfaA+LC. A site-directed mutagenesis was carried out to eliminate a *Bst*B1 restriction site within the LC to allow for subsequent cloning work. The GAP promoter in each construct was then replaced FragB_0052. A unique restriction site (FragB_0052 (HindIII)+LC) was introduced in the promoter ahead of the light chain transgene to allow for linearization of the final vector prior to *K.phaffii* transformation. The plasmid carrying the light chain was then digested using the enzyme *Xma*I and *Bam*HI while that carrying the heavy chain was digested using *Xma*I and *Bgl*II. The fragments carrying the light chain and the heavy chain respectively were ligated to obtain the final expression vector pPROMOTER+ Fab-3H6. The resulting vector was linearised at the unique restriction site and 5 µg of the linearised plasmid was transformed in *K.phaffii* X33 by DTT-Method-Electroporation method. The cells were spread on YEPD agar plates (selection 100 µg/mL zeocin) and incubated for 3-4 days at 30°C. The transformants were cultures in 96-well plates for 2 days (100 µl of YEPD+ Zeocin) and ELISA assays were carried out on the culture supernatants to verify for expression of Fab-3H6 fragments. Following confirmation by ELISA, the expressing clones were restreaked onto YEPD agar + zeocin to generate single colonies. Genomic DNA was extracted from the single-copy clones and PCRs were carried out to verify the orientation and site of integration of the respective plasmids into the genome.

For the construction of Human Lysozyme (HuLy) expressing clones, wild-type HuLy was amplified from the pAOX:Huly *K.phaffii* clone used in Hesketh *et al*. (Hesketh et al., 2013). The final vector pPICZαA-Promoter+Huly was constructed by deleting the pAOX:mating-factor in pPICZαA vector and replacing it with the constitutive promoter + mating-factor alpha fragment + HuLy cassette. Expression vectors were thereby prepared by cloning the HuLy transgene in-frame with the mating-factor alpha coding sequence at the *Xho*1/*Not*1 restriction site of the plasmid. The plasmid was linearised prior to being transformed into the X33 strain and transformants selected on a 25-100 µg/mL range of zeocin concentrations. The transformants were then cultured in 96-well plates for 3 days before assaying culture supernatants for human lysozyme activity. Genomic DNA was extracted from the clones that proved positive for human lysozyme production and used for PCR to verify the site and orientation of the integrated plasmids in the genome. Primer sequences are provided in Table 1.

Table 1. Primer sequences used in this study

| Primers for qPCR (copy number) | HuLy_F | TTGGCTAAGTGGGAATCTGG |
| --- | --- | --- |
|  | HuLy_R | CGTTTAGCACAAGCAACAGC |
|  | Fab HC_F | AGCAGAGCCATGGAAAGAG |
|  | Fab HC_R | CCGTACCCAATTGACGTTCT |
|  | Fab LC_F | CTGTTGTGTGCCTGCTGAAT |
|  | Fab LC_R | GACTTCGCAGGCGTAGACTT |
| Primers for locus check | GAPUp_F | TCTTTCATCGGCACATTTCA |
|  | GAPGene_R | CAGCTCTGGAAAGAGGCGACT |
|  | Chr1-4_0586Up_F | CAAGGTGAGTCCAGCCATTT |
|  | Chr1-4_0586Gene_R | GTTGGCTCTGGGCAGTAAGT |
|  | FragB_0052Up_F | TCCAACTCGTGTCAGATTGC |
|  | FragB_0058Gene_R | CGCTTGTCAATACCACCACA |

**Identification of significantly changing fluxes**

For each case, 1000 random values for each flux in the distribution were generated such that each value remained within bounds of the allowable range for that flux, which was determined through flux variability analysis. The Mann–Whitney *U* test was applied to those values for each individual flux separately, in order to identify those changes that had appreciable biological impact, the average of the 1000 values for each individual flux were employed in fold-change analyses. Flux values lower than 10^−4^ were set to zero. Flux Scanning based Enforced Objective Function (FSEOF)(Choi et al., 2010) was employed by constraining the r-protein production reaction to 0, 0.05, 0.1, 0.15, 0.2 and 0.25 mmol/gDW/h and the reactions whose flux values increased as the flux going through the r-protein production reaction was increased were identified.

**References**

Choi HS, Lee SY, Kim TY, Woo HM. 2010. In silico identification of gene amplification targets for improvement of lycopene production. *Appl. Environ. Microbiol.* **76**:3097–105.

Hesketh AR, Castrillo JI, Sawyer T, Archer DB, Oliver SG. 2013. Investigating the physiological response of Pichia (Komagataella) pastoris GS115 to the heterologous expression of misfolded proteins using chemostat cultures. *Appl. Microbiol. Biotechnol.* **97**:9747–9762.
