# Supplementary material for: Process development for the continuous production of heterologous proteins by the industrial yeast, Komagataella phaffii
Source: Biotechnol Bioeng. 2018 Oct 24;115(12):2962–73. doi: 10.1002/bit.26846 (PMC6283250; doi:10.1002/bit.26846)
Supplement: Supplementary file 6 — Supporting information [file BIT-115-2962-s006.docx]

Supp. File 6

Fig. S1. Growth characteristics under best performing condition at mid-exponential phase.

Table S1. Remaining substrate levels at mid-exponential and stationary phase under best performing condition

|  |  | Wild type | | HuLy | | Fab-3H6 | |
| --- | --- | --- | --- | --- | --- | --- | --- |
|  |  | Glucose | Glycerol | oSPI1 | GAP | Tef1-α | GAP |
| remaining glucose (g/L) | mid-exponential | 15.31  ±1.67 | n/a | n/a | 18.56  ±1.60 | 16.09  ±1.25 | 17.81  ±3.87 |
|  | stationary phase | nd | n/a | n/a | nd | nd | nd |
| remaining glycerol (g/L) | mid-exponential | n/a | 24.08  ±0.27 | 22.51  ±0.71 | n/a | n/a | n/a |
|  | stationary phase | n/a | nd | nd | n/a | n/a | n/a |
| remaining sorbitol (g/L) | mid-exponential | 8.91  ±0.61 | 8.26  ±0.69 | 6.74  ±1.15 | 6.89  ±1.59 | 6.85  ±2.34 | 8.65  ±1.42 |
|  | stationary phase | nd | 0.04  ±0.015 | 0.02  ±0.02 | 0.06  ±0.01 | 0.04  ±0.01 | 0.03  ±0.02 |
| remaining ammonia (g/L) | mid-exponential | 2.70  ±0.35 | 3.07  ±0.37 | 2.67  ±0.39 | 2.92  ±0.44 | 2.26  ±0.37 | 2.59  ±0.27 |
|  | stationary phase | 0.095  ±0.001 | 0.094  ±0.002 | 0.094  ±0.001 | 0.09  ±0.001 | 0.092  ±0.01 | 0.096  ±0.0002 |
| OD (600 nm) | mid-exponential | 2.27  ±0.155 | 1.78  ±0.057 | 2.12  ±0.092 | 2.47  ±0.067 | 1.96  ±0.032 | 1.94  ±0.068 |
|  | stationary phase | 54.3  ±6.359 | 58.57  ±0.764 | 55.2  ±0.954 | 38.3  ±2.858 | 39.53  ±3.265 | 41  ±5.724 |

*n/a indicates not applicable and nd indicates not detectable
