# Supplementary material for: Process development for the continuous production of heterologous proteins by the industrial yeast, Komagataella phaffii
Source: Biotechnol Bioeng. 2018 Oct 24;115(12):2962–73. doi: 10.1002/bit.26846 (PMC6283250; doi:10.1002/bit.26846)
Supplement: Supplementary file 7 — Supporting information [file BIT-115-2962-s007.docx]

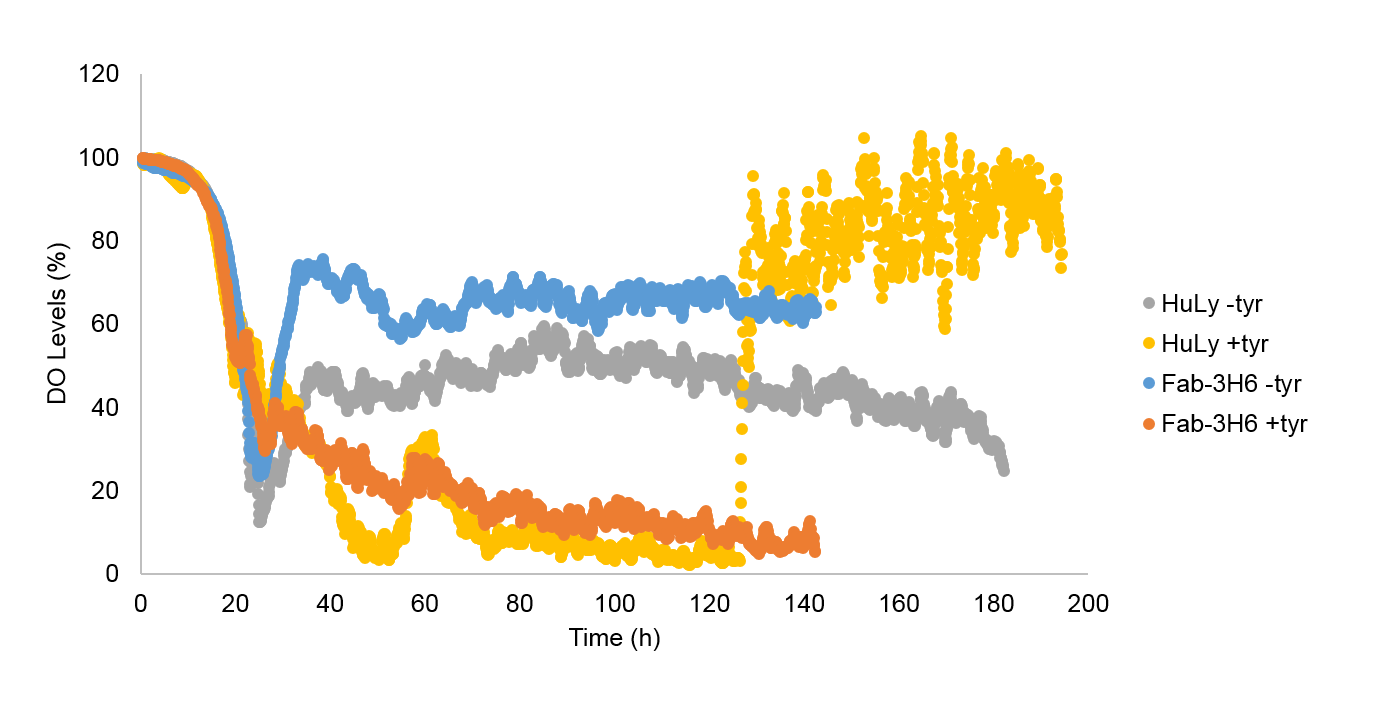


Supp. File 7

Fig. S2. DO levels of the chemostat experiments conducted with HuLy and Fab-3H6 expressing strains with and without tyrosine supplementation.

The cultures were switched to chemostat mode from batch cultivation at around t=24h. In the case of the Huly-expressing strain under tyrosine-supplemented conditions (+tyr), DO control was initiated at t=127h; dissolved oxygen, from then on, was maintained at >= 50% saturation.
