# Supplementary material for: Process development for the continuous production of heterologous proteins by the industrial yeast, Komagataella phaffii
Source: Biotechnol Bioeng. 2018 Oct 24;115(12):2962–73. doi: 10.1002/bit.26846 (PMC6283250; doi:10.1002/bit.26846)
Supplement: Supplementary file 8 — Supporting information [file BIT-115-2962-s008.docx]

**Supporting Information**

Supplementary File 1. Additional supplementary methods.

Supplementary File 2. 12 different medium compositions used to test the productivity of the strains of interest.

Supplementary File 3. Kp. 1.1., the stoichiometric model of the Komagataella phaffii metabolic network, which includes the reactions for the synthesis of Lysozyme.

Supplementary File 4. The lists of reactions that were identified to be significantly and differentially changed and associated with higher flux values with increased r-protein production, and the list of genes associated with those reactions.

Supplementary File 5. The list of the genes that were identified as being in the top 5 most-expressed genes in both the pre-induction and post-induction steady states across all 3 strains.

Supplementary File 6. The Figure represents the remaining substrate levels at mid-exponential phase for wild-type strain grown using glucose (blue) or glycerol (orange) as the carbon source, HuLy producing strain under oSPI1 (grey) and GAP (yellow) promoters, Fab-3H6 producing strain under the TEF1-α (dark blue) and GAP (green) promoters.

Supplementary File 7. The Figure represents the dissolved oxygen levels (DO %) of the growth medium in chemostat cultures during experiments conducted to test the effect of tyrosine supplementation on the productivity of the HuLy and Fab-3H6 producing strains.
